# Enzalutamide Versus Abiraterone as a First-Line Endocrine Therapy for Castration-Resistant Prostate Cancer: Protocol for a Multicenter Randomized Phase 3 Trial

**DOI:** 10.2196/11191

**Published:** 2018-07-27

**Authors:** Isao Hara, Shimpei Yamashita, Satoshi Nishizawa, Kazuro Kikkawa, Toshio Shimokawa, Yasuo Kohjimoto

**Affiliations:** ^1^ Department of Urology Wakayama Medical University Wakayama Japan; ^2^ Clinical Research Center Wakayama Medical University Wakayama Japan

**Keywords:** castration resistant prostate cancer, abiraterone, enzalutamide, prostatic neoplasms, castration-resistant, clinical protocols

## Abstract

**Background:**

Recent large-scale randomized studies have demonstrated that 2 new hormone preparations (abiraterone and enzalutamide) prolong survival in docetaxel-treated or -naïve castration-resistant prostate cancer patients. However, no studies have directly compared antitumor effects between these 2 agents, and no clear guidelines are available for choosing between them.

**Objective:**

The objective of this clinical study is to compare antitumor effects and adverse events between abiraterone and enzalutamide by allocating castration-resistant prostate cancer patients deemed not indicated for docetaxel treatment to receive either of the 2 agents.

**Methods:**

This study is an open-label, comparative study allocating castration-resistant prostate cancer patients to abiraterone or enzalutamide treatment arms (allocation factors: age <70 vs ≥70 years, and presence vs absence of metastases) and assessing the treatment results. Each arm will contain 25 patients. On confirmation of prostate-specific antigen failure or progression on imaging, patients undergo crossover to receive the alternative study drug. The primary end point is prostate-specific antigen response rate (percentage of patients with a decrease in prostate-specific antigen level by ≥50%) in the abiraterone and enzalutamide treatment arms.

**Results:**

Recruitment started in May 2016, and 13 patients have been recruited so far. We expect to complete enrollment by December 2020.

**Conclusions:**

Recently, cross-resistance between abiraterone and enzalutamide has been an issue of focus. Urologists thus tend to prefer docetaxel rather than sequential therapies using 2 hormonal preparations after the progression of a first hormonal preparation. From that perspective, our clinical trial is rather out of fashion. Nevertheless, we assume that many patients receive hormonal sequential therapy in the actual clinical setting, since most such patients cannot receive chemotherapeutic agents due to old age or poor performance status. This is why we are attempting this randomized clinical trial comparing abiraterone versus enzalutamide. We will try to identify which drug is suitable for initial hormonal therapy among castration-resistant prostate cancer patients who do not meet the indications for docetaxel therapy in terms of not only antitumor effect, but also adverse events and quality of life.

**Trial Registration:**

University Hospital Medical Information Network UMIN000022102; https://upload.umin.ac.jp /cgi-open-bin/ctr_e/ctr_view.cgi?recptno=R000025463 (Archived by WebCite at http://www.webcitation.org/70xaQfGlJ)

## Introduction

Hormone therapies are considered beneficial for the treatment of advanced prostate cancer [1]. In fact, hormone therapies are known to be safe and highly effective, but the biggest drawback is the lack of sustained antitumor effects [1]. Scientists have long been frustrated in attempts to find pharmacotherapies, including anticancer agents, that would prolong survival among patients with prostate cancer that has acquired resistance to hormone therapies (castration-resistant prostate cancer [CRPC]).

In 2004, docetaxel became the first anticancer agent confirmed to prolong survival in CRPC patients in 2 large-scale clinical trials [2,3] and was adopted as the first-line treatment for CRPC in Japan. In addition, recent large-scale randomized studies have demonstrated that 2 new hormone preparations (abiraterone and enzalutamide) prolong survival in docetaxel-treated [4,5] or -naïve CRPC patients [6,7]. Clinical use of the 2 agents in Japan began in 2014. While these 2 hormone preparations (abiraterone and enzalutamide) have different mechanisms of action, both exhibit strong inhibitory effects on the remaining androgen after androgen-deprivation therapy, leading to antitumor effects. However, to our knowledge, no studies have directly compared antitumor effects between these 2 agents, and clear guidelines are lacking for choosing between them.

The objective of this clinical study is to compare antitumor effects and adverse events between abiraterone and enzalutamide by allocating CRPC patients deemed to not meet the indications for docetaxel treatment to receive either of the 2 agents.

## Methods

### Study Patients

#### Inclusion Criteria

First, participants in the study are CRPC patients after concomitant antiandrogen therapy with at least a single agent who are docetaxel naïve and deemed to not meet the indications for docetaxel treatment (regardless of the presence or absence of metastasis). CRPC is defined as an increase in prostate-specific antigen (PSA) that is 25% or more and 2 ng/mL (2 μg/L) or more over the nadir PSA level obtained on measurements taken at least 4 weeks apart, with the day of CRPC confirmation defined as the day of relapse (day of disease progression). Testosterone level is measured at the same time to confirm that the level is no higher than the castration level (ie, <50 ng/dL or 1.7 nmol/L). The first-line therapy will be a docetaxel-prednisolone therapy for patients with a Gleason score of 8 or higher and multiple bone metastases for whom the duration of response to a hormone therapy is short (approximately standard, within 2 years) and whose systemic conditions can more than withstand docetaxel-prednisolone therapy.

Second, participants’ absolute PSA level is 5 ng/mL (5 μg/L) or higher. Third, participants are less than 85 years of age. Fourth, participants have an Eastern Cooperative Oncology Group Performance Status of 0 to 2 and expectation of survival 3 months or longer. Fifth, participants have normal organ function, with a white blood cell count of 3000/mm^3^ or greater or a neutrophil count of 1500/mm^3^ or greater; aspartate aminotransferase (AST) and alanine aminotransferase (ALT) concentrations less than 1.5 times the institutional upper limit; and serum creatinine concentration less than 1.5 times the institutional upper limit. Sixth, participants have personally provided written informed consent to participate in the study.

#### Discontinuation Criteria

If discontinuation of the study drug administration is deemed justified due to a serious adverse event or when any of the following criteria are met, the investigator shall discontinue study drug administration at their discretion and record in the case report form the reasons for discontinuation and the findings available at discontinuation: (1) study continuation is deemed difficult due to disease progression (PSA recurrence or clinical recurrence); (2) study continuation is deemed difficult due to an adverse event; (3) grade 4 toxicities have occurred as assessed by the Japanese Common Terminology Criteria for Adverse Events (CTCAE) version 4.0 by the Japan Clinical Oncology Group and Japan Society of Clinical Oncology [8]; (4) the participant or a family member has withdrawn consent or requested to discontinue the study treatment; (5) study continuation is deemed difficult due to an unforeseen incident; or (6) study continuation is deemed difficult by the investigator due to any other reason.

#### Dose-Reduction Criterion

In cases of mild adverse events, the dose of the study drug may be halved and then gradually increased or decreased depending on the condition of the participant.

### Study Design

**Figure 1:**
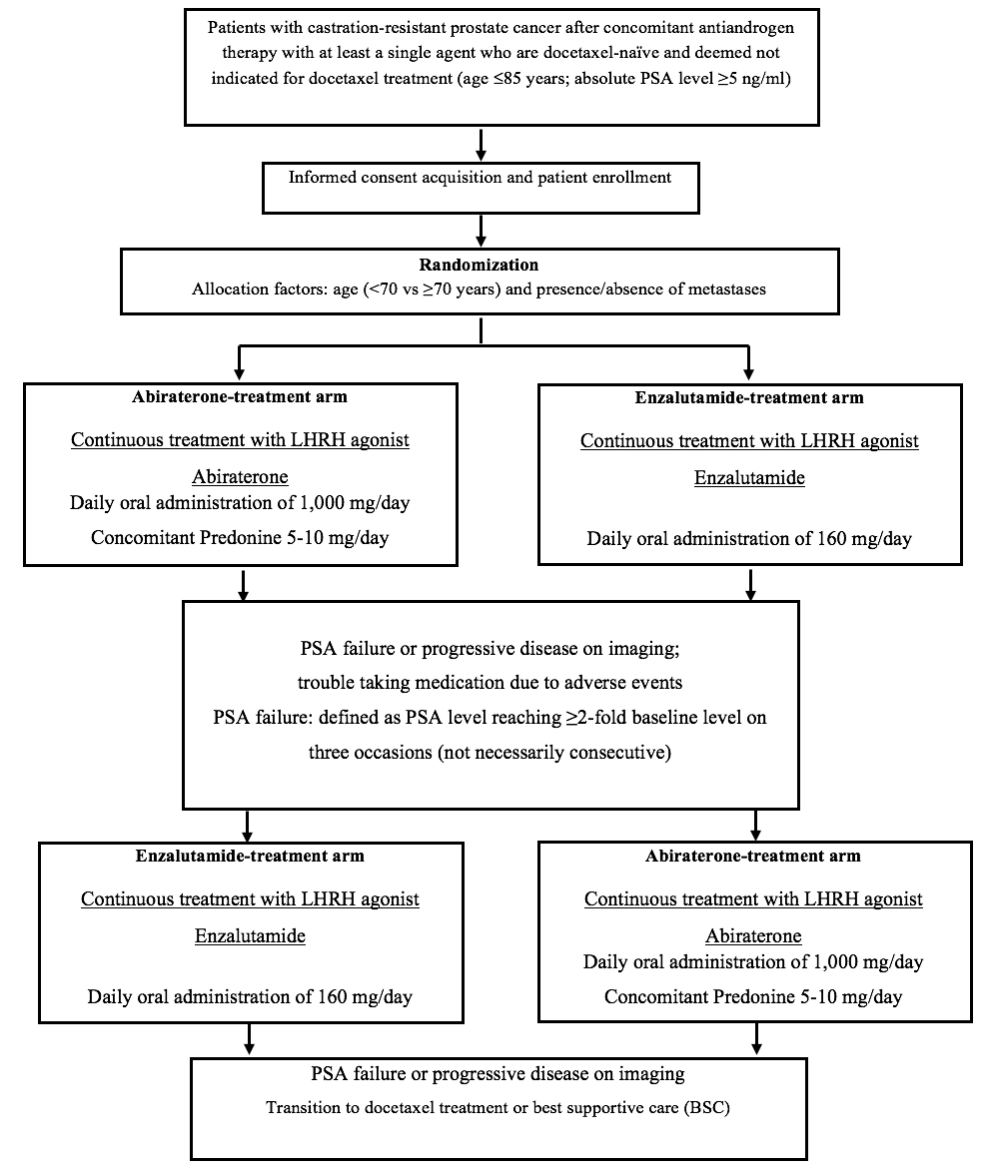
Clinical trial flowchart. LHRH: luteinizing hormone-releasing hormone; PSA: prostate-specific antigen.

The study is an open-label, randomized comparative study that allocates CRPC patients to an abiraterone treatment arm or an enzalutamide treatment arm (allocation factors: age <70 vs ≥70 years, and presence vs absence of metastases) and will assess the treatment results. Figure 1 shows a flowchart of the study design.

### Patient Enrollment

The patient enrollment center is at the Clinical Research Center, Wakayama Medical University.

#### Enrollment Procedure

The investigator checks that candidate patients conform with the inclusion criteria and enrolls patients according to the following procedure. (1) Acquire written consent from the patient, complete the required information on a patient enrollment sheet, and transmit the sheet by facsimile to the patient enrollment center. (2) The patient enrollment center checks the information provided on the patient enrollment sheet for eligibility, enrolls the patient, and allocates the patient to a treatment arm. The patient enrollment center sends the investigator a patient enrollment notification that provides information on the allocated treatment. (3) The investigator checks the patient enrollment notification sent by the patient enrollment center and initiates administration of the allocated treatment drug.

#### Random Allocation and Stratification Factors Used for Allocation

The patient enrollment center randomly allocates patients to either the abiraterone treatment arm or the enzalutamide treatment arm in a 1:1 ratio. We use the factors of (1) age (<70 vs ≥70 years) and (2) presence or absence of metastases as stratification factors for random allocation.

### Treatments

#### Hormone Therapy

Surgical castration or treatment with a luteinizing hormone-releasing hormone agonist (either leuprorelin acetate or gosererin acetate) continues even after diagnosis of CRPC.

#### Study Drugs

##### Abiraterone

Abiraterone (Zytiga 250 mg tablets; Janssen Pharmaceutica NV, Beerse, Belgium) is a prostate cancer therapeutic agent (cytochrome P450 [CYP] 17 inhibitor). Usually, for adults, it is administered in a dose of 1000 mg orally as abiraterone acetate, once daily under fasting conditions concomitantly with prednisolone. The precautions related to dosage and administration are as follows. (1) Food causes an increase in the maximum concentration and area under the curve for abiraterone. As a result, abiraterone should not be taken from 1 hour before to 2 hours after a meal (refer to the Pharmacokinetics section of the patient package insert). (2) The researcher administering prednisolone should be familiar with the information in the Clinical Results section of the patient package insert. (3) In cases of elevated values for liver function tests while a patient is undergoing abiraterone treatment, abiraterone should be temporarily interrupted, reduced in dose, or discontinued with the following guidelines as a reference.

###### Adverse Drug Reactions

Until the time of approval, adverse drug reactions (ADRs; including laboratory abnormalities) occurred in 46 of 95 patients (48.4%) evaluated for safety in the Japanese phase 2 clinical study. Major ADRs were increased AST in 13 patients (13.7%), increased ALT in 12 patients (12.6%), hypokalemia in 8 patients (8.4%), hyperlipidemia in 7 patients (7.4%), and hypertension in 4 patients (4.2%; unpublished data).

In phase 3 clinical studies conducted outside of Japan, ADRs (including laboratory abnormalities) occurred in 991 of 1333 patients (74.3%) evaluated for safety. The major ADRs were fatigue in 328 patients (24.6%), hot flash in 202 patients (15.2%), hypokalemia in 188 patients (14.1%), nausea in 179 patients (13.4%), peripheral edema in 160 patients (12.0%), hypertension in 125 patients (9.4%), constipation in 108 patients (8.1%), diarrhea in 101 patients (7.6%), vomiting in 92 patients (6.9%), dizziness in 81 patients (6.1%), increased AST in 69 patients (5.2%), and increased ALT in 68 patients (5.1%) [4,7].

###### Clinically Significant Adverse Drug Reactions

As cardiac failure and other serious cardiac disorders may occur (frequency unknown), patients are closely monitored. In the event of any abnormality, appropriate actions need to be taken, including discontinuation of treatment.

Fulminant hepatitis may occur (unknown frequency). Moreover, hepatic function disorder accompanied by increased AST (13.7%), increased ALT (12.6%), or increased bilirubin (2.1%) may occur and may result in hepatic failure (unpublished data). Thus, patients must be monitored closely using periodic liver function tests. In the event of any abnormality, appropriate actions such as dose reduction or treatment interruption or discontinuation need to be taken.

Hypokalemia accompanied by symptoms such as convulsion or muscular weakness may occur, with some cases reportedly resulting in arrhythmia. Patients should be monitored closely by periodic measurements of serum electrolyte concentrations, including serum potassium. In the event of any abnormality, appropriate actions such as potassium supplementation or interruption of abiraterone treatment should be taken.

Thrombocytopenia may occur (unknown frequency), so patients must be closely monitored. In the event of any abnormality, appropriate actions including interruption of abiraterone treatment should be taken.

As rhabdomyolysis may occur, any muscular weakness, myalgia, increased creatine kinase (or creatine phosphokinase), and increased myoglobin in the blood or urine should be noted. In the event of any such symptoms, appropriate actions including discontinuation of abiraterone treatment should be taken.

##### Enzalutamide

Enzalutamide (Xtandi 40 mg capsules; Astellas Pharma, Tokyo, Japan) is a prostate cancer therapeutic agent. Usually, for adults, it is administered in a dose of 160 mg orally as enzalutamide, once daily. The efficacy and safety of enzalutamide have not been established in patients without concomitant surgical or medical castration. The precautions related to dosage and administration are as follows. It must be administered with care to the following patients: (1) patients with a current or past history of epilepsy or other convulsive disease (convulsive seizure may occur), and (2) patients predisposed to convulsive seizure (eg, patients complicated with cerebral injuries or stroke, who have such a history, or who are undergoing treatment with an agent that lowers the convulsive seizure threshold). Important precautions are as follows: (1) as an agent for endocrine therapy, enzalutamide should be used only in patients deemed indicated for enzalutamide treatment by a physician who is well versed and experienced in pharmacotherapies for cancer, and (2) as convulsive seizure may occur, patients undergoing treatment with enzalutamide should exercise caution when operating a motor vehicle or other machines associated with potential hazards.

###### Drug Interactions

Enzalutamide is metabolized mainly by the drug-metabolizing enzyme CYP2C8. Moreover, enzalutamide exhibits induction effects on CYP3A4, CYP2C9, CYP2C19, CYP2B6, uridine diphosphate-glucuronyltransferase, and P-glycoprotein. It exhibits inhibitory activity against P-glycoprotein, breast cancer-resistance protein, organic cation transporter 1, and organic anion transporter 3 [9]. Due to the long elimination half-life (4.7-8.4 days), enzalutamide may still induce or inhibit metabolic enzymes and transporters after completion of treatment.

In Japanese phase 1 and 2 clinical studies in CRPC patients, 31 of 47 patients who received enzalutamide (66.0%) developed ADRs. Major ADRs included hypertension (14.9%), constipation (14.9%), fatigue (12.8%), decreased appetite (12.8%), decreased weight (10.6%), and prolonged QT on electrocardiograms (10.6%; unpublished data; as of the time of approval of the drug in March 2014.)

In phase 3 studies conducted outside of Japan in CRPC patients with prior docetaxel treatment, 554 of 800 patients who received enzalutamide (69.3%) developed ADRs. Major ADRs included fatigue (21.5%), nausea (20.1%), hot flash (15.0%), decreased appetite (12.6%), and asthenia (10.0%; as of the time of approval of the drug in March 2014) [5]

In an international phase 3 clinical trial in chemotherapy-naïve CRPC patients, 556 of 871 patients (including 28 Japanese) who received enzalutamide (65.0%) developed ADRs. Major ADRs included fatigue (25.3%), hot flash (13.4%), and nausea (13.3%; as of the time of amendment of precautions related to indications, October 2014) [6]

###### Clinically Significant Adverse Drug Reactions

Frequencies of the following ADRs are based on the tabulation of patients who received enzalutamide in the Japanese phase 1 and 2 clinical studies, non-Japanese phase 3 clinical studies, and an international phase 3 clinical trial.

As convulsive seizure (frequency 0.2%) such as convulsion and status epilepticus may occur, patients are monitored closely. In the event of any abnormality, treatment should be discontinued and appropriate actions taken.

Thrombocytopenia may occur (frequency unknown). As decreased platelets may occur, patients are monitored closely. In the event of any abnormality, treatment should be discontinued and other appropriate actions taken.

#### Definitions

The protocol treatment period is defined as the period of study drug (abiraterone or enzalutamide) administration as primary or secondary treatment.

The study period is defined as the period from the day of consent until the day of confirmation of the final outcome.

The investigator shall survey the outcome in patients after the protocol treatment is stopped until outcome confirmation or loss to follow-up.

#### Allocation to Study Drug

We are enrolling patients after obtaining written informed consent from each patient after providing them with written information on details of the study.

Patients are allocated to a treatment arm (abiraterone or enzalutamide treatment arm) in such a way that both arms are comparable in terms of (1) age (<70 vs ≥70 years) and (2) presence versus absence of metastases.

#### Study Schedule

Table 1 shows the study schedule. Tests listed in Table 1 are conducted on each patient before enrollment to assess whether the patient meets the inclusion criteria. Blood tests, including PSA, are conducted monthly. A quality-of-life survey (Functional Assessment of Cancer Therapy-Prostate [FACT-P]) is conducted every 3 months. Computed tomography (CT) bone scintigraphy is performed every 6 months.

Blood tests, including performance status and PSA, CT (chest and abdomen, plain), bone scintigraphy, and other tests are performed at crossover to the alternative treatment or when symptoms lead to suspicion of disease progression.

**Table 1:** Study schedule.

Test	Screening	Every month	Every 3 months	Every 6 months	At crossover or suspected disease progression	At study completion
Informed consent	+	–	–	–	–	–
Participant characteristics and underlying disease information	+	–	–	–	–	–
Eastern Cooperative Oncology Group performance status	+	+	–	–	+	–
Prostate-specific antigen	+	+	–	–	+	–
Hematology tests	+	+	–	–	+	–
Biochemical tests	+	+	–	–	+	–
Computed tomography (chest and abdomen, plain)	+	–	–	+	+	–
Bone scintigraphy	+	–	–	+	+	–
Prior treatment and concomitant therapy	+	–	–	–	–	–
Quality of life (Functional Assessment of Cancer Therapy-Prostate)	+	–	+	–	–	–
Adverse event	–	+	–	–	+	–
Information on discontinuation	–	–	–	–	+	–
Final outcome	–	–	–	–	–	+

#### Crossover of Study Drugs

On confirmation of PSA failure (deemed to have occurred when the PSA level reaches double the baseline level on 3 occasions, not necessarily consecutive, or progression is evident on imaging or a patient is considered to have trouble taking the study drug due to adverse events), the patient crosses over to receive the alternative study drug. Participants in this clinical study are those for whom docetaxel is not considered to be indicated. Nevertheless, before a patient crosses over to receive the alternative study drug, we explain to the patient again about the appropriateness of docetaxel treatment and evaluate the appropriateness. Patients who are deemed indicated for docetaxel treatment at this point in time shall be considered a dropout and given docetaxel treatment.

### Observations, Tests, and End Points

#### Before Treatment Initiation

Before a patient initiates treatment, we check their consent to participate, performance status, PSA, hematology tests, biochemical tests, CT (chest and abdomen, plain), bone scintigraphy, presence or absence of prior treatment, and quality of life.

#### Efficacy Evaluation

PSA is checked monthly. Plain CT of the chest and abdomen and bone scintigraphy are performed every 6 months. At treatment crossover (enzalutamide to abiraterone, abiraterone to enzalutamide) or a switch to another therapy, including best supportive care, or when symptoms indicate suspected progression of disease stage, we perform plain CT of the chest and abdomen and bone scintigraphy as well, so as to assess the effects of the prior treatment and to check for any new lesions.

The measurement of PSA or imaging of progression-free survival with respect to the primary treatment begins on the first day of the primary treatment. The measurement of overall PSA or imaging progression-free survival after crossover from the primary to secondary treatment also begins on the first day of primary treatment. The measurement of time to use of chemotherapy or best supportive care, or the overall survival, also begins on the first day of the primary treatment.

#### Safety Evaluation

##### Adverse Events

An adverse event is any event (abnormal clinical finding, subjective or objective symptom, or abnormal change in laboratory test value) occurring during the study after initiation of the study drug treatment, regardless of the relationship to the study drug.

We evaluate adverse events based on an abnormal finding, symptom, laboratory test value, or severity according to the CTCAE version 4.0 [8].

##### Serious Adverse Events

An adverse event is serious if the event is observed any time after initiation of the study drug treatment up to 30 days following treatment completion (or discontinuation) and (1) results in death, (2) is life-threatening, (3) requires hospitalization or prolongation of hospitalization for treatment, (4) results in persistent or significant disability or incapacity, or (5) results in a congenital anomaly.

On the occurrence of a serious adverse event for which a causal relationship to the protocol treatment cannot be excluded, the investigator shall provide appropriate interventions or treatments, promptly (within 24 hours of awareness) complete the required information on a Serious Adverse Event Report in accordance with the procedure at the medical institution the investigator is affiliated with, and communicate the event to the study secretariat by facsimile.

##### Laboratory Tests

Hematology tests are white blood cell count, differential white blood cell counts, hemoglobin, and platelet count. Tests are conducted at baseline and once monthly thereafter.

Clinical chemistry tests are AST, ALT, alkaline phosphatase, total bilirubin, creatinine, albumin, sodium, potassium, chlorine, phosphorus, and calcium. Tests are conducted at baseline and once monthly thereafter.

#### Primary End Point

The primary end point is PSA response rates (percentages of patients with PSA level decreasing by ≥50%) to the primary treatment in the abiraterone and enzalutamide treatment arms.

#### Secondary End Point

Secondary end points are (1) PSA or imaging progression-free survival with the primary treatment in the abiraterone and enzalutamide treatment arms, (2) PSA response rate (percentage of patients with PSA level decreasing by ≥50%) with secondary treatment in the abiraterone and enzalutamide treatment arms, (3) overall PSA or imaging progression-free survival after crossover treatment with both abiraterone and enzalutamide, (4) time to use of chemotherapy or best supportive care, (5) overall survival, (6) comparison of quality of life as assessed by FACT-P, and (7) adverse events.

### Ethical Considerations

#### Compliance Regulations

All researchers involved in this research study shall comply with the *Declaration of Helsinki* (seventh revision, October 2013 [10], as translated by the Japan Medical Association) and the *Ethical Guidelines for Medical and Health Research Involving Human Subjects* [11] (enforced on April 1, 2015 and partially revised on February 28, 2017) in the conduct of this research.

#### Informed Consent

The principal investigator or investigator shall fully inform each participant by written information to allow them to decide whether to participate in the study and shall obtain from each participant written informed consent to participate in the study based on their own free will.

On obtaining written informed consent, the principal investigator or investigator who informed the participant shall confirm whether the participant fully understood the contents of the written information before consenting. The principal investigator or investigator shall fill in the date on which the information was provided and the date of confirmation of the participant’s intent on the written informed consent form and affix their seal or signature to the form. Each participant shall provide consent after gaining a full understanding of the contents of the written information, and shall then affix their seal or signature to and date the form.

The principal investigator or investigator shall provide a copy of the sealed or signed written informed consent along with the written information to the participant who provided consent and shall properly retain the original of the written consent at their medical institution.

When a matter arises that concerns the individual’s intent to participate in the study, the principal investigator or investigator shall amend the written information, inform the participant again using the revised written information, and obtain written informed consent from the individual to continue participation in the study based on their own free will.

In the event a participant in the study requests withdrawal of consent, the request is documented in a study participation withdrawal form. If possible, a consent withdrawal form should be prepared. The participant shall fill in the date of withdrawal of consent and affix their seal or signature to the consent withdrawal form, and the principal investigator or investigator shall fill in the date of verification and affix his or her seal or signature to the form. The principal investigator or investigator shall provide a copy of the sealed or signed consent withdrawal form to the individual who withdraws consent and shall retain the original at their medical institution.

#### Approval by Institutional Review Board or Ethics Committee

Before the study is underway, the protocol, written information for the patient, written informed consent form, and the justification to conduct the study must be submitted for review by a committee at each study site (such as an institutional review board or ethics committee pursuant to the regulations of the study site) and receive its approval.

#### Safeguard of Personal Information

All parties involved in this study shall strictly safeguard the personal information of participants pursuant to the Japanese Personal Information Protection Act. When providing case report forms or information on adverse events and other relevant data to a party outside of his or her own medical institution, the investigator shall pay due attention to safeguarding personal information by actions such as replacing the identities of the participants concerned with participant identification codes or enrollment numbers so that no third party can identify the individuals. A reference table should be prepared that links the enrollment numbers issued to each participant at the time of acquisition of consent to each of their fields of personal information (name and medical record number), to allow identification or collation, as necessary, of enrolled participants whose information has been anonymized. The reference table is retained under strict safeguard at each study site. When identifying or collating an enrolled participant, the enrollment number issued at enrollment is used. Similar measures are taken to safeguard the personal information of participants when publishing results of this research.

#### Important Findings on Genetic Characteristics

We do not expect this research to yield any important findings on the genetic characteristics of participants that may be relevant to health or inherited by their offspring.

#### Compensation

In the event the conduct of this study causes any adverse events that result in health hazards to a participant, the investigator shall administer appropriate treatments and take the best possible actions, including other necessary measures. Furthermore, as the conduct of this study is covered by insurance, any health hazards will be handled within the scope of the Adverse Drug Reaction Relief System [12].

#### Remuneration or Financial Burden to Study Participants

This research does not provide any remuneration to or impose any financial burden on the study participants.

#### Disclosure of Participant Information and Handling of Inquiries from Participants

In the event a participant personally requests the disclosure of information that involves privacy issues, in principle, the researchers (the principal investigator, coordinator, and investigator) at the study site that enrolled the participant shall handle such a request.

Participants may submit general inquiries or file complaints related to privacy by postal mail or email to the first author.

### Statistics

#### Analysis Sets

The full analysis set includes all enrolled patients, excluding those with any major protocol violations (did not provide consent or any major procedural violations).

The per-protocol set includes those participants in the full analysis set who receive the study treatment allocated according to the protocol, excluding those who do not meet any eligibility criteria, meet any exclusion criteria, or take any prohibited concomitant drugs or other agents.

In all efficacy evaluations, we use the full analysis set as the primary analysis set, with analyses of the per-protocol set performed for reference purposes. We use the per-protocol set for safety evaluations.

#### Efficacy Evaluation

##### Primary End Point

For the primary end point, we calculate the point estimate and Clopper-Pearson exact 80% CI of the PSA response rate with primary treatment among the full analysis set population in the abiraterone and enzalutamide treatment arms, respectively. We determine the 95% CI and perform Fisher exact test for reference purposes. Furthermore, we calculate the odds ratio and 95% CI for PSA response. We estimate the impacts of prognostic factors and treatment effects by multiple logistic regression analysis, and estimate odds ratios adjusted based on the regression coefficient of a multiple logistic regression analysis and its 95% CI. We use adjustment factors for allocation and any patient characteristics distributed unevenly between arms (with *P* ≤.2 as a guide) as influencing factors.

##### Secondary End Points

For PSA or imaging progression-free survival with primary treatment in the abiraterone and enzalutamide treatment arms, we determine the estimated survival curve among the full analysis set population in each arm by the Kaplan-Meier method. Under certain circumstances, we perform a similar analysis on the per-protocol set population. In such cases, we use the Greenwood formula to calculate the 95% CI and determine median survival, 1-year survival rate, and respective CIs. In addition, we use the Cox proportional hazard model to estimate the impacts of prognostic factors and treatment effects. We determine the estimated hazard ratio and its 95% CI based on the regression coefficient of the Cox proportional hazard model. We use adjustment factors for allocation and any participant characteristics that were distributed unevenly between arms (with *P* ≤.2 as a guide) as prognostic factors.

For PSA response rates with secondary treatment in the abiraterone and enzalutamide treatment arms, we determine rates by performing an analysis similar to that used to determine PSA response rates with the primary treatment among the full analysis set population in the abiraterone and enzalutamide treatment arms.

For overall PSA or imaging progression-free survival after crossover of the 2 treatment arms (abiraterone and enzalutamide), we determine survival by performing an analysis similar to that used to determine the PSA or imaging progression-free survival with the primary treatment among the full analysis set population in the abiraterone and enzalutamide treatment arms.

For time to initiation of chemotherapy or best supportive care, we calculate medians and interquartile ranges among the full analysis set population in the abiraterone and enzalutamide treatment arms and perform comparative analysis by the Wilcoxon test.

We determine overall survival by performing an analysis similar to that used to determine the PSA or imaging progression-free survival with the primary treatment among the full analysis set population in the abiraterone and enzalutamide treatment arms.

For comparison of quality of life as measured by FACT-P, we calculate medians and interquartile ranges among the full analysis set population in the abiraterone and enzalutamide treatment arms and perform a comparative analysis by the Wilcoxon test.

We tabulate adverse events in each arm and compare the severity and frequency of adverse events between arms.

### Target Sample Size

Our target sample size is 50 participants (n=25 per arm). With no crossover treatment regimen consisting of abiraterone and enzalutamide, as first-line plus second-line treatment, available in Japan, we plan to conduct a pilot parallel-arm study in this research. As such, the primary end point selected is the proportion of participants with a PSA response 50% or greater with first-line treatment. In studies to date, the PSA response with abiraterone as prior treatment was 78% in the PREVAIL study [6] investigating abiraterone as first-line treatment and enzalutamide as second-line treatment, while the PSA response was 62% in the COU-AA-302 study [7] investigating enzalutamide as first-line treatment and abiraterone as second-line treatment. Given its nature as a pilot study, the research must necessarily evaluate PSA responses in both arms in Japan, assuming that patient characteristics are evenly distributed. We thus consider CI of 80% (confidence coefficient .80) by the Clopper-Pearson exact method for the PSA response in each arm. With the expected PSA response of 70% in both arms and assuming a 1-sided interval width of 0.15%, the minimum sample size required is 21. Allowing for the potential that a few participants would become ineligible, we selected a sample size of 25 per arm, or a total of 50. The confidence coefficient of .80 is considered to correspond to a significance level of .10 on the 1-sided alternative hypothesis in a single arm. PSA response with second-line treatment was 17.6% for enzalutamide (first-line treatment) and abiraterone (second-line treatment) and 22.9% for abiraterone (first-line treatment) and enzalutamide (second-line treatment; Nadal et al [13]). With the expected PSA response with second-line treatment at 20% and assuming a sample size of 25, the 1-sided width of the 80% CI is 12.8%, which allows for estimation based on a CI width equivalent to that for the PSA response with first-line treatment.

### Protocol Changes And Study Discontinuation or Completion

#### Protocol Changes

In the event of protocol changes becoming necessary during the study, the principal investigator shall decide what changes to make and promptly inform the investigator at each study site in writing about the changes and the reasons thereof. In cases of significant change to the protocol, the investigator shall report the change to the head of the medical institution and obtain an approval for the change, along with approval from the institutional review board or ethics committee.

#### Completion of Protocol

Once data lock is confirmed, we will consider the study to be complete. On receiving communication of the data lock from the data center, the principal investigator shall report on completion of the study to the investigator at each study site, who shall report the completion to the head of the medical institution and the ethics committee.

#### Discontinuation of Protocol

The rules to discontinue the entire study are as follows: (1) when the principal investigator determines after evaluating reports of study progress and study monitoring that completing the study is difficult due to reasons such as patient enrollment delays or frequent protocol deviations; (2) when serious safety or efficacy issues are judged to be associated with the study to justify its discontinuation based on new information that has become available after initiation of the study; and (3) when it is determined that safety issues are associated with the study or that continuation of the study is not meaningful based on an evaluation of relevant information obtained from sources outside of this study, such as literature articles or conference presentations.

The procedure for deciding to discontinue the entire study are as follows. The principal investigator must request the ethics committee to conduct a review and accept its recommendations. Based on the recommendations, the principal investigator shall make a determination on the necessity to discontinue the entire study according to the rules provided in the preceding subsection. If the principal investigator disagrees with the recommendations, then the principal investigator shall report the reasons to the ethics committee.

After making a decision to discontinue the entire study, the principal investigator shall communicate with the investigators immediately about the reasons thereof and what actions to take. On receiving such a communication, investigators shall inform participants about discontinuation of the entire study and the reasons thereof, and shall immediately take appropriate actions.

### Study Control

#### Monitoring

Monitors shall make sure that the human rights, safety, and welfare of participants are protected and that this study is conducted in compliance with the most up-to-date protocol and standard operating procedures. In addition, monitors shall access source documents and other study-related records directly to confirm that the data and other information reported by the principal investigator or investigators are accurate and complete.

#### Monitoring Methods

The data center shall perform monitoring centrally and periodically. In central monitoring, the case report forms collected and other data reported are checked to make sure that the study is conducted in a safe manner and in accordance with the protocol. Monitoring results are to be submitted to the principal investigator and ethics committees.

#### Deviations From Per-Protocol Treatments

The principal investigator or investigator may deviate from the protocol for this study if such deviation is medically unavoidable so as to avoid immediate hazard to a participant. In such a case, the principal investigator or investigator shall report the deviation and the reasons thereof to the ethics committee through the head of his or her institution as soon as possible. Moreover, the principal investigator or investigator shall document all deviations from the protocol for this study, regardless of the reasons.

#### Audits

No audits are planned in this study.

### Retention of Source Documents and Other Records

#### Scope of Source Documents

The term source document in the study refers to any of the following: (1) records pertaining to participant consent and information provision; and (2) medical records, laboratory test data, imaging study films, and other records on which case report form data are based; data saved in electronic medical records are also considered source documents.

#### Retention of Records by Participating Medical Institutions

The principal investigator shall retain the following study-related records for 5 years from the date of the final report or 3 years from the date of the final publication of the study results, whichever is later: (1) source documents; (2) informed consent forms and other documents related to this study or copies thereof that have been prepared by the personnel of a participating medical institution; (3) protocol (the latest version), documents pertaining to study review obtained from the institutional review board, and other documents obtained during the conduct of the study; and (4) other documents generated in the work related to the study.

#### Disposal of Records

Records will be disposed of according to the methods and procedures for retention and disposal established by the institution with which the researcher is affiliated, with consideration given to methods such as anonymization.

### Information Entries in Case Report Forms and Their Submission

The investigators and others taking part in this study shall prepare case report forms in accordance with the *Guide on Filling Out Case Report Forms* (internal document) and submit the prepared case report forms to the data center by postal mail or in person. Investigators and other taking part shall prepare and retain a copy of each case report form before its submission.

### Study Period

The study runs from December 2014 to December 2023. The patient enrollment period is September 2014 to December 2020.

### Data Publication

The principal investigator has registered this study with the University Hospital Medical Information Network Clinical Trials Registry (UMIN000022102) before beginning the study and shall register the end-of-study results on the same registry after study completion. Furthermore, the principal investigator shall publish the results in a conference presentation or a thesis promptly after completion of the study. Author names and their order shall be approved by the principal investigator and the protocol author before publication of the results in any conference presentation or journal submission.

### Party Responsible for Costs of Study (Source of Funding)

As this study is supported by the research fund of the Department of Urology, Wakayama Medical University, Wakayama, Japan, there are no conflicts of interest.

## Results

Recruitment started in May 2016, and 13 patients have been recruited so far. We expect to complete enrollment by December 2020.

## Discussion

In terms of sequential therapy of novel hormonal preparations (abiraterone and enzalutamide), clinical outcomes of enzalutamide following abiraterone [14,15] or abiraterone following enzalutamide [16,17] have been reported. However, these were rather small-scale retrospective studies and, to our knowledge, no randomized trials comparing these sequential therapies have been reported.

Recently, cross-resistance between abiraterone and enzalutamide has been an issue of some focus. The most well-known mechanism of resistance is AR-V7, which is an androgen receptor splicing variant [18]. Patients expressing AR-V7 showed lower response rates and poor prognosis when treated with abiraterone as well as enzalutamide. In fact, the response rate to the second drug is reportedly lower than that to first therapy in sequential therapies [14-17]. Urologists thus tend to prefer docetaxel over sequential therapies using 2 hormonal preparations after the progression of the first hormonal preparation. From that perspective, our clinical trial is rather out of fashion. Nevertheless, we assume that many patients are forced by necessity to undergo sequential hormonal therapy in the actual clinical setting, since most such patients cannot receive chemotherapeutic agents due to old age or poor performance status. This is why we are undertaking this randomized clinical trial comparing abiraterone versus enzalutamide. We are trying to identify which drug is most suitable for the initial hormonal therapy (in terms of not only antitumor effects, but also adverse events and quality of life) among CRPC patients who do not meet the indications for docetaxel therapy.

## Abbreviations

ADR: adverse drug reaction
ALT: alanine aminotransferase
AST: aspartate aminotransferase
CTCAE: Common Terminology Criteria for Adverse Events
CRPC: castration-resistant prostate cancer
CT: computed tomography
CYP: cytochrome P450
FACT-P: Functional Assessment of Cancer Therapy-Prostate
PSA: prostate-specific antigen
